# A Case Report of Madelung’s Disease in Romania

**DOI:** 10.3390/diagnostics15040459

**Published:** 2025-02-13

**Authors:** Andrei Ionut Cucu, Anca Sava, Amelian Madalin Bobu, Claudia Florida Costea, Vlad Liviu Hartie, Emilia Patrascanu, Laurentiu Andrei Blaj, Catalin Mihai Buzduga, Ana Maria Dumitrescu, Camelia Tamas, Ana Cristina Istrate, Otilia Boisteanu

**Affiliations:** 1 Faculty of Medicine and Biological Sciences, University Stefan cel Mare of Suceava, 720229 Suceava, Romania; andrei.cucu@usm.ro; 2 Emergency Clinical Hospital Prof. Dr. Nicolae Oblu, 700309 Iasi, Romania; claudia.costea@umfiasi.ro (C.F.C.); liviu.vlad.hartie@umfiasi.ro (V.L.H.); laurentiu-andrei.blaj@d.umfiasi.ro (L.A.B.); istrate.ana-cristina@d.umfiasi.ro (A.C.I.); 3 Department of Morpho-Functional Sciences I, Faculty of Medicine, University of Medicine and Pharmacy Grigore T. Popa Iasi, 700115 Iasi, Romania; anna.dumitrescu91@gmail.com; 4 St. Spiridon County Clinical Emergency Hospital Iasi, 700111 Iasi, Romania; amelian.bobu@gmail.com (A.M.B.); catalinbuzduga@gmail.com (C.M.B.); camelia6ta@yahoo.com (C.T.); otilia.boisteanu@umfiasi.ro (O.B.); 5 Department of Ophthalmology, University of Medicine and Pharmacy Grigore T. Popa Iasi, 700115 Iasi, Romania; 6 Department of Anesthesia and Intensive Care, University of Medicine and Pharmacy Grigore T. Popa Iasi, 700115 Iasi, Romania; patrascanu.emilia@umfiasi.ro; 7 Regional Institute of Oncology, 700483 Iasi, Romania; 8 Department of Endocrinology, University of Medicine and Pharmacy Grigore T. Popa Iasi, 700115 Iasi, Romania; 9 Department of Plastic Surgery, University of Medicine and Pharmacy Grigore T. Popa Iasi, 700115 Iasi, Romania

**Keywords:** Madelung syndrome, Launois–Bensaude syndrome, benign symmetric lipomatosis

## Abstract

**Background:** Madelung’s disease is a rare lipid metabolic disorder characterized by diffuse and symmetrical adipose tissue deposits in the subcutaneous fascial spaces, presenting with multiple painless masses throughout the body. The disease is more common in middle-aged adults with a history of chronic alcohol consumption. **Case Report:** This article reports a case of Madelung’s disease from Romania in a 67-year-old man admitted to our department for multiple adipose masses located in the neck and upper back. MRI examination of the head and neck revealed symmetrical and non-encapsulated fat deposition. Surgical intervention was performed to resect the adipose masses. The article also discusses the etiology, clinical manifestations, diagnosis, and surgical treatment of large adipose lesions. **Conclusions:** This case report provides insights for the diagnosis and treatment of Madelung’s syndrome.

## 1. Introduction

Madelung disease (MD), also known as multiple symmetric lipomatosis, benign symmetric lipomatosis, or Launois–Bensaude syndrome, is a rare lipid metabolic disorder characterized by diffuse adipose tissue deposition in the superficial and deep subcutaneous fascial spaces, slow tumor growth, and progressive enlargement [1,2]. The fat deposits are symmetrical and non-encapsulated, unlike typical lipomas. They arise on the patient’s body, usually around the neck, shoulders, upper trunk, or limbs [3].

The disease is more common in Mediterranean countries and primarily affects middle-aged males, with a male-to-female ratio of 15:1 [4,5]. Exceptional cases have also been reported in children [6]. The neck, shoulders, back, and proximal upper and lower limbs are regions where fatty masses most commonly develop, but the literature has also reported unusual locations such as the tongue [7,8,9], orbit [10], perineum [11], and scrotum [12].

This disease was first described in 1846 by Sir Benjamin Brodie [13,14]. Later, in 1888, German surgeon Otto Wilhelm Madelung presented a series of 35 patients with “cervical lipomatosis.” Ten years later, in 1898, Launois and Bensaude [15] described another 64 patients with this syndrome, characterized by excessive adipose tissue around the neck, shoulders, and trunk. Subsequently, MD also became known as Launois–Bensaude syndrome [16]. In 1970, Greene et al. reported that, among the European authors, 150 cases had identified [17].

Enzi [18] was the first to propose a classification of MD based on the anatomical distribution of adipose masses. In MD-Type I, the fat masses are symmetrically located predominantly around the neck (known as Madelung’s collar or “horse collar”), shoulders, supraclavicular fossa, and proximal upper limbs. In Type II, the fat deposits are located in the abdomen and thighs, potentially leading to confusion with normal obesity. In this Type II variant, the neck and upper trunk regions are not affected [19].

Subsequently, Donhauser et al. proposed a more recent classification [20], which expanded on Enzi’s types: type 1––distribution of masses in the neck region (fatty neck type); type 2––pseudo-athletic appearance (pseudo-athletic type); and type 3––gynecoid type [20]. Some patients may exhibit more than one type, such as type 1+2, type 1+3, type 2+3, or even all three types combined [19]. Building on the hypothesis that the proliferative process begins in the neck and spreads caudally, Schiltz et al. proposed in 2018 a reclassification of MD into five phenotypes [21].

Currently, more than 400 cases of MD have been reported in the worldwide medical literature [1]. Although most cases occur sporadically, the number of reported cases has increased in recent years [2,22]. The majority of MD patients have been reported from Europe, particularly from southern countries such as Portugal and Italy. In Asia, most cases have been reported in South Korea and China [2,23].

This article analyzes the clinical data of a 67-year-old male patient from Romania with MD and discusses the etiology, clinical manifestations, diagnosis, and treatment strategy for such cases.

## 2. Case Report

### 2.1. Clinical Data

The 67-year-old normoweight patient was admitted to the Department of Neurosurgery at Prof. Dr. Nicolae Oblu Hospital, Iași, with massive thickening of the neck and upper back. The progressive enlargement of these tumor-like masses had been observed over several years. From the patient’s medical history, it was noted that he suffered from poorly controlled arterial hypertension and was a moderate alcohol consumer. No symptoms were reported until discomfort, including aesthetic concerns, prompted the patient to seek medical advice. The patient had no history of dyspnea or dysphagia.

**Figure 1:**
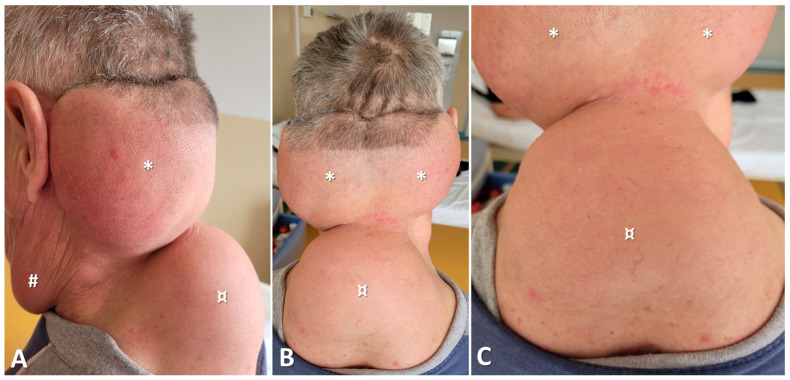
(**A**) Left lateral cervical view highlighting the lipomatous masses: a bulky mass located in the anterior neck region (#), the posterior occipito-cervical region (*), and the cervico-dorsal region (¤); (**B**) Posterior view showing two masses in the occipito-cervical region (*) and one mass in the cervico-dorsal region (¤); (**C**) Posterior cervico-dorsal view (detailed).

Physical examination revealed four soft, painless masses with poorly defined margins. The first tumor-like mass was located in the anterior neck region and had a soft consistency. Two firm-elastic masses were located in the occipito-cervical region, extending laterally over the neck, surpassing the mastoid processes, and covering the sternocleidomastoid muscles. The lower boundary of these masses corresponded to the third cervical vertebra. The fourth mass, also firm-elastic, was located in the cervico-thoracic region, with the upper boundary at the level of the C3 vertebra and the lower boundary at the fifth thoracic vertebra, giving the clinical appearance of a “buffalo hump” (Figure 1).

**Figure 2:**
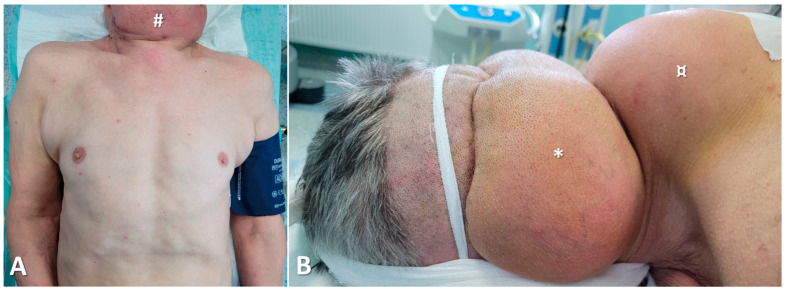
(**A**) Anterior view showing the lipomatous mass in the anterior neck region (#) and trunk lipomatosis; (**B**) Lateral view highlighting the masses located in the occipito-cervical region (*) and the cervico-dorsal region (“buffalo hump”) (¤).

Additionally, physical examination revealed lipomatosis in the trunk region, giving a pseudo-athletic appearance (Figure 2).

### 2.2. Imaging Studies

**Figure 3:**
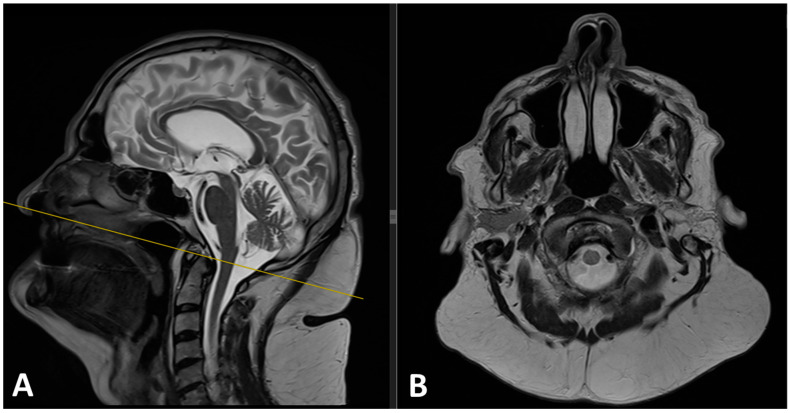
(**A**) Preoperative sagittal T2 and (**B**) axial T2 MRI images revealed significant hypertrophic fat tissue around the neck and upper back, consistent with the diagnosis of Madelung disease.

A head and neck MRI exam, performed using a 1.5 Tesla scanner (MAGNETOM Sola, syngo MR XA51, Erlangen, Germany), confirmed the presence of well-defined, non-encapsulated subcutaneous fat tissue with bilateral and symmetric distribution, showing a normal hyperintense signal in both T1 and T2-weighted sequences. To exclude signs of malignancy, a contrast agent was also administered. The tumor-like masses were located in the anterior neck region, the occipital region, the posterior cervical region, and the upper back (Figure 3).

**Figure 4:**
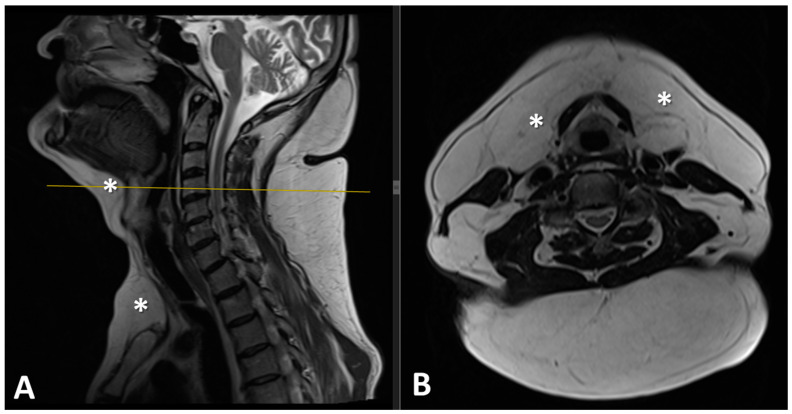
(**A**) Preoperative sagittal T2 and (**B**) axial T2 MRI images revealed significant soft-tissue mass of high-signal intensity with low-signal intensity fibrous septa at the level of the chin, submandibular, and suprasternal region (*).

The MRI examination of the neck revealed the presence of adipose deposits in the submentonian, submandibular, and suprasternal regions (Figure 4).

### 2.3. Surgical Treatment

After admission to our department, all preoperative examinations were completed. The neurological examination ruled out the compression of neurovascular structures or other neurological conditions. The cardiac examination only identified essential arterial hypertension, which the patient was not managing properly due to non-compliance with treatment. Laboratory tests showed no significant abnormalities, nor were any other related diseases identified that might cause MD. After discussions with the patient and family, the final decision was made to remove all the posterior cervical and upper back lipomas through surgery. The patient underwent a surgical procedure under general anesthesia, with orotracheal intubation, which proceeded without complications.

**Figure 5:**
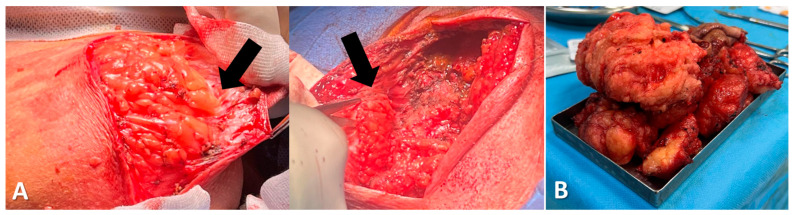
(**A**) Surgical findings: it was observed that the lipoma lacks a capsule, with no cleavage plane between the tumor and the adjacent anatomical structures (*black arrows*); (**B**) Postoperative specimen: yellow adipose tissue, soft and solid, with an envelope in some areas, and lobulated.

Two oblique incisions were made along the posterior cervical masses, while a midline incision was performed for the mass located in the upper back. During the surgery, cervical arteries and veins were carefully protected. Additionally, during the procedure, we observed multiple septated lipomatous accumulations of fatty tissue adhering to neighboring structures with poorly defined edges. The absence of a capsule around the lipoma made the dissection particularly difficult (Figure 5A).

The adipose tissue was highly vascularized, which made hemostasis difficult throughout the surgical procedure. Surgical excision was carefully carried out, and a large area of hyperplastic fatty tissue (Figure 5B) was successfully removed. After resection, external drainage tubes were placed. The tumor was excised and sent to the pathology department for examination. Postoperatively, anti-inflammatory medications and supportive symptomatic treatment were administered. The drainage tube was removed after 5 days. The patient’s wound healed well, without the accumulation of exudate, redness, or swelling of the surrounding skin. The patient was safely discharged in stable condition.

**Figure 6:**
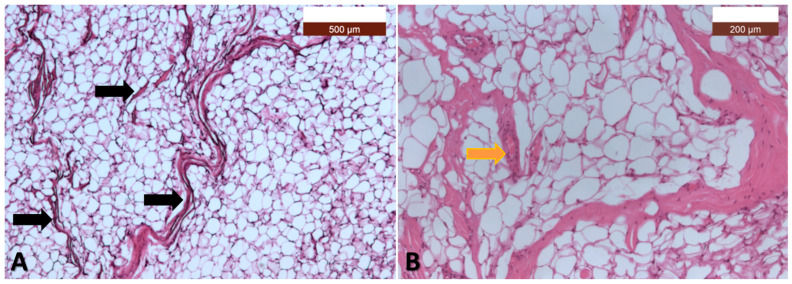
(**A**) Adipose tissue not delimited by a capsule, intersected by numerous connective septa of varying thicknesses, lengths, and orientations (*black arrows*). The adipose tissue consists of mature adipocytes, but with varying shapes and sizes (HE, ×5); (**B**) With a higher magnification (HE, ×10), it is observed that the connective septa are numerous and contain blood vessels of the arteriole type (*yellow arrow*) (HE, ×10).

**Figure 7:**
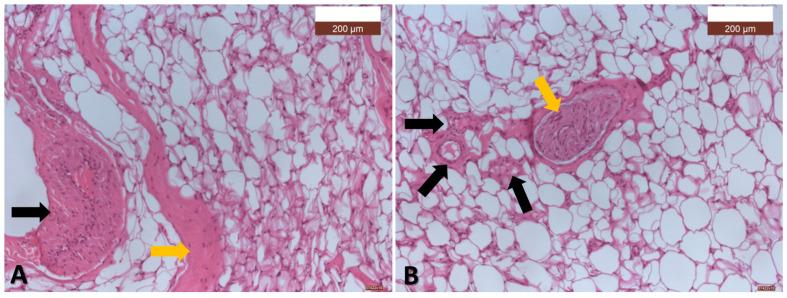
(**A**) The adipose tissue, composed of mature adipocytes with varying shapes and sizes, is intersected by simple, densely cellularized connective septa (*orange arrow*) and connective septa containing muscular-type arteries (*black arrow*) (HE, ×10); (**B**) The adipose tissue, composed of mature adipocytes with varying shapes and sizes, is intersected by numerous short connective septa containing capillaries or arterioles (*black arrows*), which appear to separate the cells from one another. Additionally, there is a longer and thicker septum within which a nerve fiber is observed (*orange arrow*) (HE, ×10).

The pathological examination identified diffusely proliferating, mature, well-differentiated adipocytes without atypia, which was consistent with a lipoma. The pathological examination confirmed the diagnosis of MD (Figure 6 and Figure 7).

**Figure 8:**
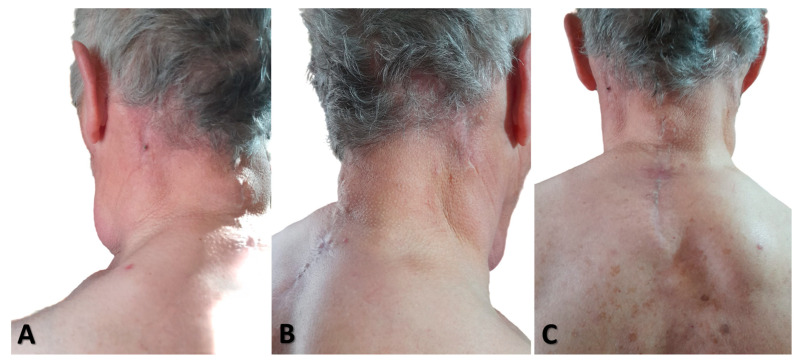
(**A**) Left lateral cervical view; (**B**) right lateral cervical view; (**C**) posterior view, showing the surgical outcomes one year after liposuction. No recurrence was observed.

There was no recurrence during the 1-year follow-up, and the patient’s appearance improved (Figure 8).

## 3. Discussion

Regarding Romania, although several individual cases [24,25,26,27] or series of patients [28,29,30,31] with MD have been reported so far, the exact incidence of the disease in our country is still not precisely known.

Currently, MD is classified into type I, type II, and type III [3,32,33]. Type I primarily occurs in men, and the diseased adipose tissue is mainly located in the neck, submentum, shoulders, upper back, and upper arm regions [1]. Type II has a similar incidence in both women and men, with adipose tissue primarily accumulating in the upper back, shoulders, buttocks, and upper thighs [34]. Type III is a congenital accumulation of fat mainly around the trunk. In this case, the patient had type I MD, with fat accumulation on both sides of the neck, occiput, submandibular region, and upper back. This corresponds with data from the literature, which reports type I of MD as the most commonly encountered form [2].

Although genetic malformations have been reported in MD patients [35], MD does not appear to be a hereditary disease. However, Payne [36] considers this possibility especially since the literature contains a few cases of familial MD, which suggest an autosomal dominant mode of inheritance [37]. Nevertheless, most authors consider MD to be a sporadic disease [38]. In this case, no one in the patient’s family showed signs of MD or the presence of any lipomas.

### 3.1. Etiology

Regarding the etiology of MD, several factors have been implicated, including chronic alcohol consumption, metabolic syndromes such as hyperuricemia, hyperlipidemia, or diabetes, liver disease, hypothyroidism, and renal tubular acidosis [2,39,40,41,42], all of which contribute to the accelerated proliferation of the disease. Paradoxically, in MD, the serum concentration of HDL cholesterol is usually high [43,44]. Although the pathogenesis of MD is still unclear, its occurrence has been associated with adipose tissue mitochondrial dysfunction, catecholamine-induced fat deposition, decreased cytochrome C oxidase activity, and reduced inducible nitric oxide synthase activity [45].

Most authors report that the primary cause of MD is long-term heavy alcohol consumption. In a review that included 286 patients with MD, Liu et al. observed that most patients exhibited similar behaviors, such as long-term alcohol abuse in 89.5% of cases and a history of smoking in 53% of cases [2]. Although the causal relationship between alcohol and MD remains unclear, Mevio et al. noted an association with chronic alcoholism in 69–90% of patients [46]. Studies have shown that long-term heavy alcohol consumption leads to mitochondrial DNA mutations, premature oxidation of mitochondrial DNA, and ultimately, mitochondrial cell dysfunction [1,47]. Several authors have reported mutations in the *LIPE*, *LMNA*, *CAPSL*, and *MFN2* genes, which are associated with energy imbalance and the accumulation of dysfunctional mitochondria [48,49,50,51]. Additionally, chronic alcohol consumption can result in a decrease in the activity and number of beta-adrenergic receptors, and consequently, the synthesis of triacylglycerols [1,52]. Furthermore, ethanol increases cytochrome P450 activity in adipose tissue, ultimately leading to apoptosis and inflammation of the adipose tissue [53,54].

Despite the existence of multiple hypotheses, such as lipolytic pathway defects caused by catecholamines, mitochondrial DNA mutations or deletions, the exact etiology and the pathophysiological pathways leading to the development of the disease remain unclear [55,56]. Although all these factors, including alcohol consumption, smoking, and obesity, are not proven as definitive determinants, authors still recommend the cessation of alcohol consumption [2].

### 3.2. Pathology

Regarding the pathological aspects of the disease, studies have shown that the diseased tissue in MD does not represent a hypertrophy of pre-existing adipose tissue but rather a proliferation of adipose cells [1]. This fact was supported by Kodish et al., who demonstrated that various stimulation and loading tests with somatotropic hormone, cortisol, insulin, or glucan led to the inhibition of free fatty acid release from lipomatous tissue in response to physiological stimuli [28,57]. They found that this occurs despite normal levels of lipolytic hormones, leading Kodish and his collaborators to suggest that this is due to the functional denervation of the diseased adipose tissue [57]. Therefore, Kodish et al. suggest that functional sympathetic denervation results in the hypertrophy of brown fat [57], especially since the distribution and type of adipocytes in MD are similar to brown adipose tissue [47]. However, from a histological perspective, the adipose tissue deposits are indistinguishable from mature fat, except for varying degrees of fibrosis and, rarely, calcification [58]. Additionally, the adipocytes in MD are smaller in size, and there is an increased content of vascular and fibrous tissues compared to normal levels [22].

More recent pathophysiological investigations have observed disruptions in lipid metabolism at the mitochondrial level, identifying multiple pathological subsarcolemmal accumulations of mitochondria in the striated muscle fibers of subjects with MD. Additionally, Southern blot tests have revealed multiple deletions of mitochondrial DNA [35].

Some authors have reported the occurrence of head and neck cancers, particularly hypopharynx squamous cell carcinoma, in patients with MD [5]. However, considering common carcinogenic risk factors (alcohol and smoking), these factors are likely responsible for the development of these tumors.

Regarding the spontaneous malignant transformation of MD, Tizian et al. described the emergence of an intramyxoid sarcoma after six years of follow-up [59], while other authors have documented a few rare cases of transformation into liposarcoma in the literature [60,61]. Since lipomas are non-encapsulated, and therefore, lipectomy or liposuction cannot achieve complete removal, recurrence after surgical treatment of lipomas is common [62].

### 3.3. Clinical Presentation

MD is characterized by the appearance of multiple, painless masses distributed all over the body, which are diffuse and symmetrical. Due to the highly complex anatomical structure of the maxillofacial region, the neck and face areas are the most affected, and in these cases, the recurrence rate is high [63]. The literature has reported that when a neck mass appears, patients may develop symptoms such as limited neck rotation, compression of the airways, or dysphagia. If the adipose tissue invades the parotid gland, patients can develop facial deformities. Invasion of the floor of the oral cavity may restrict tongue movements and cause dysphagia. Additionally, invasion of the tongue can result in the development of a “mega-tongue” [64].

In the case of MD, patients seek medical assistance mainly due to aesthetic deformities and mobility limitations, as well as peripheral neuropathies or restricted movements that can impact their daily activities and quality of life. Some patients face life-threatening risks, such as tracheobronchial obstruction, dysphagia, dysphonia, mediastinal syndrome, or autonomic neuropathy [65,66,67,68]. Although MD can cause breathing difficulties, most patients seek medical attention primarily for aesthetic reasons and because it worsens their self-perception [42,69]. In our case, the patient presented to the hospital due to restricted neck movements and discomfort in a lying position caused by adipose tissue masses, as well as progressive cosmetic deformities caused by these fatty masses. This restriction in neck movement without other neurological symptoms ruled out the possibility of myasthenia gravis or muscular dystrophies, which have been described in the literature as associated conditions [70]. In our patient’s case, the adipose tissue deposits increased over a few years, and, in contrast to Decum’s disease (adiposis dolorosa), these masses were painless [71].

MD is often associated with other conditions, such as hypertension, diabetes, hyperlipidemia, hyperuricemia, as well as diseases affecting the liver, kidneys, or thyroid gland (hypothyroidism) [72]. Some authors have observed that 60–90% of patients with MD had a liver condition, such as hepatic steatosis or alcoholic liver cirrhosis [32]. Among all these factors, our patient presented with hypertension and a history of long-term alcohol consumption.

### 3.4. Diagnosis

Although some authors consider that the diagnosis of MD is based on (i) clinical manifestation, (ii) imaging examination (CT, MRI, or Doppler ultrasound), and (iii) the patient’s long-term drinking history [1], others consider histological examination to be important in the primary diagnosis of the disease [22].

Doppler ultrasound examination is currently the first-line diagnostic tool for MD [73] and highlights irregularities and diffuse thickening of the subcutaneous adipose tissue, without the presence of capsules [74]. Doppler ultrasound provides important information about the major vessels of the neck, as well as the presence of lymphadenopathy [73].

CT examination reveals the accumulation of multiple subcutaneous masses, well-defined and low-density, which compress or even deform adjacent structures [32].

MRI examination can identify the non-encapsulated deposition of fat tissue in the subcutaneous layers [73], while classic lipomas appear as soft-tissue masses with high-signal intensity and low-signal intensity fibrous septa. Due to its superior soft tissue contrast resolution, MRI exploration represents the diagnostic standard [29], allowing the evaluation of cleavage planes between lipoma, muscle, and vessels [75], which is particularly important in cases located in the neck and maxillofacial areas [29]. The use of a contrast agent is crucial when sarcomatous degeneration is suspected, as it better assesses both the tumor margins and irregular vascularization [75,76]. In conclusion, MRI exploration is valuable not only for assessment but also for preoperative planning [53]. In this case, the patient was directly evaluated with MRI, including the administration of a contrast agent, not only to obtain a diagnosis but also to confirm the absence of findings suspicious for malignancy, since cases of malignant transformation of adipose masses in MD have been reported in the literature [60,61]. Therefore, although differential diagnosis is challenging, it is important to keep in mind the potential for spontaneous transformation into liposarcoma [59,60]. In accordance with the clinical manifestations, MRI findings, and pathological results, the diagnosis of MD was accurately established in this case.

### 3.5. Treatment

The goal of treatment in MD is to restore function and improve the patient’s physical appearance [51]. Treatment options for MD include lipectomy, liposuction, or local drug injection lipolysis [77]. Among these, the first two remain the primary classical treatment options for MD, although their effectiveness remains uncertain [2]. Liu et al. reported in a review that lipectomy was the most common treatment method (69.5%, n = 308), followed by liposuction (24.2%), and a combination of liposuction and lipectomy (6.1%). In the same study, the recurrence rate was similar for both lipectomy and liposuction, with a mean recurrence time of 6.3 years (ranging from 7 months to 10 years) [2]. Additionally, recurrence was most commonly observed in the neck area, likely due to the difficulty in completely removing adipose deposits in this region [2].

Adipose tissue resection (lipectomy) is suitable for patients with larger lesions [19], while liposuction is generally recommended for those with smaller adipose masses [19]. In the case of large tumor masses, surgery is the most effective treatment, as it alleviates compression symptoms, improves local dysfunction, and restores physical appearance, thereby enhancing the patient’s quality of life [73]. Furthermore, lipectomy allows for the accurate identification and direct visualization of vital structures, as well as efficient hemostasis [19]. In this case, the resection of adipose masses was performed in a single surgical intervention. However, due to the wide range of lesions seen in these patients, the authors recommend a method of staged resection to avoid increased bleeding during the operation [3].

Liu et al. believe that, at present, the most effective treatment methods are still considered to be lipectomy and liposuction. Of course, the choice of surgical method should take into account the severity of the disease (volume of fat deposits), the location of the tumor masses, patient expectations, and the surgeon’s experience [2]. However, the majority of surgeons [19,78] prefer open lipectomy, through which adipose lumps can be safely removed under direct observation, thereby protecting tissue structures and preventing excessive bleeding [79]. Since fatty deposits are unencapsulated and diffusely infiltrate muscles and vessels without a clear dissection plane, some authors consider open surgery to be mandatory in certain anatomical regions [38]. In the case of our patient, due to the localization of lipomas in the cervical region, we opted for open surgery to visualize the vital anatomical structures, especially since liposuction is typically indicated for lipomas located in the thoracic, abdominal, or limb regions, and is rarely used for lipomas in the cervical area [26].

Regarding the subcutaneous administration of lipolytic solutions (mesotherapy), such as lecithin or phosphatidylcholine, these treatments may have beneficial outcomes. However, the most concerning side effect, neuropathy, prevents the routine use of this technique [80,81].

Patients with MD have an increased risk of difficult intubation due to extensive anterior neck lipomatosis, limited neck and cervical mobility, and a higher incidence of postoperative hematomas. In such challenging cases, awake videolaryngoscopy may be an option in managing patients with MD [65,82]. In some cases, protective tracheostomy may also be considered [19].

Another controversial issue is the placement of skin incisions, which is typically performed directly over the adipose masses. Most authors recommend a single transverse incision rather than multiple direct incisions [78]. In cases of more exposed locations, such as the neck or face, aesthetic considerations must be taken into account [19].

In the literature, the most common reported postoperative complications include anemia, temporary facial palsy, seroma, hematoma, infection, skin necrosis, and numbness [2]. These complications can occur particularly due to a blind approach to the cervical and maxillofacial areas, which are regions with rich vascularization and superficial innervation [26]. In our case, there were no postoperative complications. To prevent the occurrence of hematomas or seromas, we recommend effective hemostasis and the use of suction drains.

Upon discharge, the patient was advised to stop alcohol consumption, as some studies have shown that abstaining from alcohol can slow the enlargement of adipose masses and reduce the risk of recurrence. [53]. After one year of follow-up, the patient experienced no recurrence of the disease.

Since the incidence of MD is very low in Romania, there are still cases of missed and misdiagnosis. The differential diagnosis includes other similar conditions such as lipomatosis, angiolipoma, lipodystrophy, liposarcoma, hypercortisolism, diffuse thyroid enlargement, salivary gland disease, Frölich syndrome, Dercum’s disease, neurofibromas, and Cushing’s syndrome [72,79,83,84,85,86].

Regarding the particularities of our case, we identified the late diagnosis of the patient at the age of 67, whereas MD is a condition frequently diagnosed in middle-aged individuals. While most patients seek medical attention for aesthetic reasons due to progressive cosmetic deformities, in this case, the patient presented to the doctor because of movement limitations in the neck caused by large occipito-cervical fat masses. A minor particularity is the patient’s occasional alcohol consumption, with laboratory tests within normal limits and no liver disease, despite most authors describing MD as occurring predominantly in chronic heavy alcohol consumers.

## 4. Conclusions

This case discusses the diagnosis and treatment of MD. Given that the pathology of MD is still not fully understood, the most effective treatment for large adipose masses is surgical resection. In cases with extensive lesions, we recommend a multi-stage surgical approach, both to reduce postoperative complications and to ensure better rehabilitation.

## Data Availability

All data are reported in the text.
